# Saliva microRNA Profile in Children with and without Severe SARS-CoV-2 Infection

**DOI:** 10.3390/ijms24098175

**Published:** 2023-05-03

**Authors:** Steven D. Hicks, Dongxiao Zhu, Rhea Sullivan, Nirupama Kannikeswaran, Kathleen Meert, Wei Chen, Srinivasan Suresh, Usha Sethuraman

**Affiliations:** 1 Department of Pediatrics, Pennsylvania State University Medical Center, Hershey, PA 17033, USA; shicks1@pennstatehealth.psu.edu (S.D.H.);; 2 Department of Computer Science, Wayne State University, Detroit, MI 48201, USA; dzhu@wayne.edu; 3 Division of Emergency Medicine, Department of Pediatrics, Children’s Hospital of Michigan, Central Michigan University, Detroit, MI 48201, USA; 4 Division of Critical Care, Department of Pediatrics, Children’s Hospital of Michigan, Central Michigan University, Detroit, MI 48201, USA; 5 Population Science, Department of Oncology, School of Medicine, Wayne State University, Detroit, MI 48201, USA; 6 Department of Pediatrics, University of Pittsburgh, UPMC Children’s Hospital of Pittsburgh, Pittsburgh, PA 15224, USA

**Keywords:** COVID-19, children, miRNA, saliva, SARS-CoV-2

## Abstract

Severe acute respiratory syndrome corona virus 2 (SARS-CoV-2) may impair immune modulating host microRNAs, causing severe disease. Our objectives were to determine the salivary miRNA profile in children with SARS-CoV-2 infection at presentation and compare the expression in those with and without severe outcomes. Children <18 years with SARS-CoV-2 infection evaluated at two hospitals between March 2021 and February 2022 were prospectively enrolled. Severe outcomes included respiratory failure, shock or death. Saliva microRNAs were quantified with RNA sequencing. Data on 197 infected children (severe = 45) were analyzed. Of the known human miRNAs, 1606 (60%) were measured and compared across saliva samples. There were 43 miRNAs with ≥2-fold difference between severe and non-severe cases (adjusted *p*-value < 0.05). The majority (31/43) were downregulated in severe cases. The largest between-group differences involved miR-4495, miR-296-5p, miR-548ao-3p and miR-1273c. These microRNAs displayed enrichment for 32 gene ontology pathways including viral processing and transforming growth factor beta and Fc-gamma receptor signaling. In conclusion, salivary miRNA levels are perturbed in children with severe COVID-19, with the majority of miRNAs being down regulated. Further studies are required to validate and determine the utility of salivary miRNAs as biomarkers of severe COVID-19.

## 1. Introduction

The coronavirus disease 2019 (COVID-19) pandemic due to severe acute respiratory syndrome coronavirus 2 (SARS-CoV-2) has resulted in 103.49 million cases and 1.11 million deaths in the United States [1]. As of 2 March 2023, 15.47 million children have tested positive for the virus, representing 18% of all cases [2]. In children, this has included a spectrum of illness ranging from mild to severe disease including multisystem inflammatory syndrome in children (MIS-C) or severe respiratory illness [3,4,5,6]. Although hospitalization rates for children with the infection are lower than for adults, one in four of those hospitalized have required critical care, with 2.3% of them needing mechanical ventilation [7,8]. Timely recognition of severe illnesses is crucial for appropriate disposition and favorable outcomes. However, since early symptoms of COVID-19 are subtle and mimic other common infections, recognizing children at risk for severe disease is challenging. This is further compounded by the fact that there are limited biomarkers specific to severe infection [4,5,6,9,10].

Host–pathogen interaction can cause epigenetic changes, facilitating evasion of immune surveillance and thus causing severe infections [11,12]. Epigenetic changes can regulate immune signaling, post-translational mRNA processing and protein secretion [13,14,15]. One of the most recognized epi-transcriptional mechanisms is the activity of microRNAs (miRNAs). These noncoding RNAs regulate gene expression at the post-transcriptional level by inhibiting mRNA translation or promoting its degradation. Dysregulated miRNAs modulate several cellular processes resulting in disease [16,17,18,19], and this has been explored for prognosis in other diseases [20,21,22,23,24,25,26,27]. Recently, miRNAs that can strongly bind to certain key SARS-CoV-2 genes were identified [28]. Additionally, target sites for 28 miRNAs were found on the SARS-CoV-2 genome, suggesting that the virus may “sponge off” and down regulate immune modulating miRNAs [29]. Such altered expression of miRNAs can lead to a dysregulated host immune response, release of inflammatory cytokines and viral replication [30,31]. Several miRNAs have been investigated as biomarkers for severe COVID-19 in adults [32,33,34,35,36,37,38,39,40]. However, the role of miRNAs as a biomarker for severe SARS-CoV-2 infection in children is unknown.

Unlike blood and serum, saliva is readily available, non-invasive, associated with higher child and parent satisfaction and has a high miRNA content [41,42]. Further, miRNAs are stable in saliva and display diagnostic and therapeutic utilities in other pediatric conditions [22,23,24]. Prior studies have demonstrated the potential utility of saliva miRNAs as biomarkers for head and neck cancer, stress and even susceptibility to childhood upper respiratory infections [43,44,45]. These results suggest that saliva miRNA levels may provide unique information about inflammation and the host immune response. However, the salivary miRNA landscape in children with SARS-CoV-2 infection is unknown.

The goals of this study were to determine the salivary miRNA profile in children with SARS-CoV-2 infection at presentation and compare the expression in those with and without severe disease outcomes. We hypothesized that a subset of host miRNAs involved in inflammatory signaling would be down regulated in children with severe COVID-19. Such knowledge could be used to develop a molecular assay assessing the risk of severe disease and define novel therapeutic targets.

## 2. Results

### 2.1. Participants Characteristics

Data from 197 participants (non-severe: 152, severe: 45) were included in the analysis. The median duration of symptoms at the time of sample acquisition was 3 (2–5) days. The demographics and other characteristics of participants are given in Table 1. The average age was 7.5 (±5.9) years, and the majority were African Americans and had public health insurance.

### 2.2. Host miRNAs in Saliva

**Figure 1:**
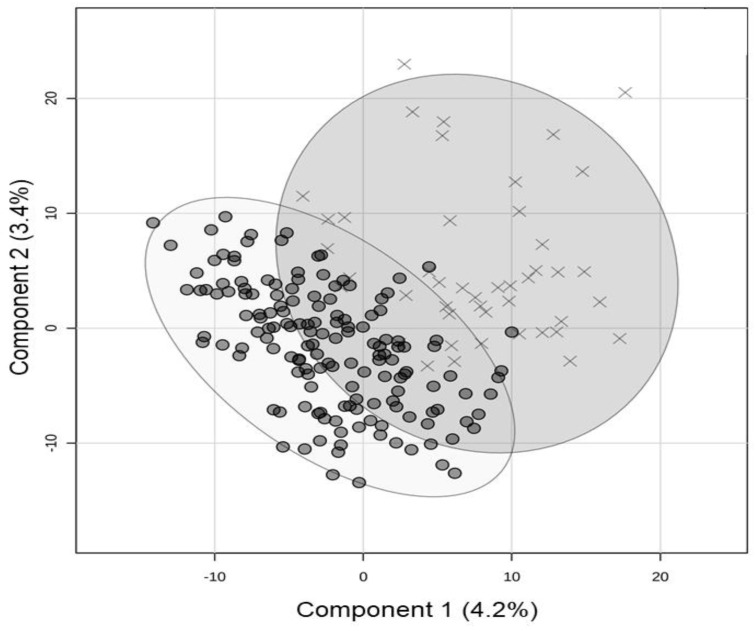
Salivary miRNA profiles in children with severe versus non-severe SARS-CoV2 illness. The two-dimensional partial least squares discriminant analysis (PLSDA) plot displays total saliva miRNA profiles for severe (X’s; *n* = 45) and non-severe (O’s, *n* = 152) cases of SARS-CoV-2, as measured with RNA sequencing. The 95% confidence intervals are shown with ovals. There was moderate separation of the two groups based on expression levels of 1606 miRNAs. However, this plot accounts for only 7.6% of the variance in miRNA data, as only 43 miRNAs displayed a significant difference between groups (defined as adjusted *p* < 0.05 and fold change > 2 on Wilcoxon Rank testing).

Of the 2652 known human miRNAs, 1606 (60%) were measured in the saliva of children with SARS-CoV-2 infection and compared between severe and non-severe groups. The mean number of total miRNA alignments did not differ (*p* > 0.05) between the severe (777,512 ± 883,625) and non-severe groups (518,666 ± 797,504). A two-dimensional PLSDA utilizing total salivary miRNA profiles achieved partial separation of severe and non-severe cases, while accounting for 7.6% of the variance between the groups (Figure 1).

### 2.3. Differential Expression of miRNAs

**Figure 2:**
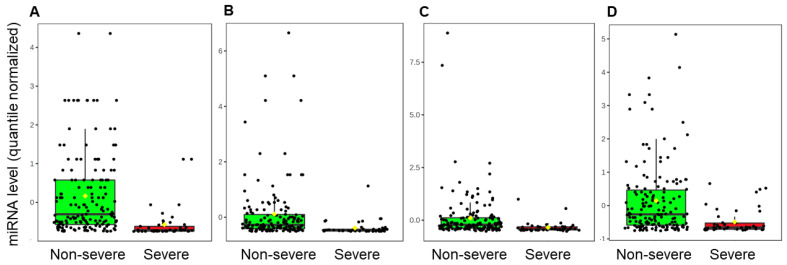
Four salivary miRNAs with large differences between severe and non-severe cases of COVID-19. The box plots represent quantile-normalized, mean-center-scaled salivary levels of miR−4495 (**A**), miR−296-5p (**B**), miR−548ao-3p (**C**) and miR−1273c (**D**) in children with severe (red; *n* = 45) and non-severe (green; *n* = 152) cases of SARS-CoV-2. All four of these miRNAs displayed a significant difference (adj *p* < 0.05, fold change > log2) on Wilcoxon Rank Sum testing. Mean (yellow diamond), median (black bar) and 95% confidence intervals are displayed.

There were 43 miRNAs that displayed ≥2-fold difference between severe and non-severe cases, while maintaining an adjusted *p*-value < 0.05 on the Wilcoxon rank sum test (Table S1—Differentially expressed miRNAs). Consistent with the sponge hypothesis, the majority of these miRNAs (31/43, 72%) were down regulated in severe cases. The largest between-group differences were observed for miR-4495 (adj *p* = 9.4 × 10^−9^, log2 fold-change = −2.36, mean read count: 3537, present in 99% of samples), miR-296-5p (adj *p* = 4.9 × 10^−7^, log2 fold change = −2.30, mean read count: 1097, present in 98% of samples), miR-548ao-3p (adj *p* = 1.0 × 10^−5^, log2 fold change = −1.66, mean read count: 153, present in 88% of samples) and miR-1273c (adj *p* = 4.6 × 10^−5^, log2 fold change = −1.67, mean read count: 384, present in 95% of samples) (Figure 2).

### 2.4. Biological Relevance to SARS-CoV-2

These four microRNAs displayed pathway enrichment for 32 gene ontology pathways (adj *p* < 0.05). (Table S2—Gene ontology pathways). Pathways with specific relevance to SARS-CoV-2 infection were viral process (adj *p* = 0.000043, 36 genes targeted by three miRNAs), transforming growth factor beta receptor signaling pathway (adj *p* = 0.018, 21 genes targeted by two miRNAs), and Fc-gamma receptor signaling involved in phagocytosis (adj *p* = 0.029, 9 genes targeted by one miRNA).

## 3. Discussion

This prospective cohort study is the first to describe the miRNA expression in saliva of children with SARS-CoV-2 infection and to our knowledge, the first to demonstrate a relationship between salivary microRNA levels and pediatric COVID-19 severity. We identified 43 miRNAs that were differentially expressed in saliva of children with severe infection compared with those with non-severe infection in the four weeks following initial diagnosis. Consistent with our hypothesis, the majority of these were down regulated. Specifically, salivary levels of four miRNAs (miR-296-5p, miR-4495, miR-548ao-3p and miR-1273c) had the largest between-group differences and displayed pathway enrichment for several gene ontology pathways including those of viral processing and phagocytosis.

Although miRNAs have been shown to impact the prognosis of other diseases [25,26,27], the literature on its role in SARS-CoV-2 infection is largely limited to laboratory or bioinformatics models with a few studies primarily in adults [28,29,32,33,34,35,36,37,38,39,40]. Farr et al. found 55 miRNAs that were altered in blood samples obtained from ten adults with COVID-19 and ten healthy controls during early-stage disease [37]. Fernandez-Pato et al. assessed the sera of 96 adults with SARS-CoV-2 infection and found 200 differentially expressed miRNAs, with upregulated putative targets of SARS-CoV-2 and inflammatory miRNAs [38]. Our study results are consistent with the above reports but are novel in their use of saliva to detect the differential expression of miRNAs in children with the infection. Obtaining saliva is easy, painless and often preferred by children and their parents, thus making it an ideal medium for a biomarker [41]. Further, it can be especially valuable in busy acute care settings, where a non-invasive, objective biomarker that predicts symptom severity could have major implications for patient care and disposition.

The down regulation of some of the host miRNAs noted in our study is consistent with previous reports in adults with COVID-19 [39,40]. The physiologic relevance of the four most down regulated host miRNAs in children with severe COVID-19 in our study is supported by their putative mRNA targets, which include transcripts involved in viral protein processing, virion assembly and immune processes including those critical for homeostasis, tissue repair and phagocytosis. Such altered expression of specific host miRNAs by a virus to regulate its replication and escape from the host immune system has also been reported with other viruses [46,47,48,49,50,51]. Specifically, the differentially expressed miR-296-5p noted in our study has been reported to be one of the key inflammatory miRNAs induced by interferons in response to viral infections [48], up regulated in human cells following enterovirus 71 (EV71) infection to prevent viral replication [49] and altered in lung cells following influenza A virus infection, where its levels are inversely correlated with key inflammatory transcripts [50]. Contrary to this, a study of individuals with human immunodeficiency virus (HIV)-1 showed that serum levels of miR-296-5p are lower compared with individuals without HIV-1 [51].

Since our study did not assess longitudinal miRNA levels in children with COVID-19, it remains unclear whether serial measurements could provide information regarding clinical trajectory and symptom recovery. Additional studies with larger sample sizes and serial measurements are required to further investigate the utility of salivary miRNAs in predicting severe COVID-19 in children.

The strengths of this study include its prospective design, two-site recruitment and relatively large sample size. However, there are several limitations that should be noted. The two recruiting sites were both large academic children’s hospitals. Hence, the results may not be generalizable to outpatient settings with a greater preponderance of mild disease. The convenience sampling may have adversely impacted our results. Since the primary focus of this study was on children who were infected with SARS-CoV-2, we did not include children without the infection as controls. Recruitment occurred between March 2021 and February 2022, when initially delta and then later omicron variants predominated, which may have impacted our results. Further, it is unclear whether these miRNA perturbations will persist with evolving COVID-19 variants. We note that saliva samples were obtained at the time of clinical presentation (median of 3 (2.5) days after symptom onset). Although we found no correlation between the levels of miRNA and the days since symptom onset, we cannot assume that measurement of levels outside this range would provide useful information regarding risk of severe illness. Similarly, follow-up to confirm the absence of severe symptoms in children with mild disease at the time of enrollment occurred on days 14 and 30. It is possible that a small portion of these children experienced relapse of severe symptoms beyond this follow-up time point. Lastly, we did not validate our findings in a separate cohort of children. These preliminary RNA sequencing results will need to be validated with quantitative real-time polymerase chain reactions in an external cohort.

## 4. Materials and Methods

### 4.1. Study Design

This was a prospective, observational study of a sample of children evaluated in the emergency departments (EDs) of two children’s hospitals in two different states between March, 2021 and February, 2022. Both hospitals are urban tertiary care centers with >70,000 annual ED visits. Both EDs had a standardized protocol for testing children for SARS-CoV-2 infection via a rapid reverse transcription polymerase chain reaction (RT PCR) test, with additional serology testing in some cases.

### 4.2. Study Definitions

Severe outcome was defined as the presence of any of the following: requirement for supplemental oxygen (≥50% FiO2), non-invasive positive pressure or mechanical ventilation, extra corporeal membrane oxygenation, vasopressors or inotropes, cardiopulmonary resuscitation or death from a related cause during hospitalization or within 1 month after discharge. Severe outcomes were determined by two investigators blinded to microRNA results via a chart review and parent survey thirty days after discharge. Any disagreements were resolved by a third investigator.

### 4.3. Participants

Children <18 years of age presenting during study hours with a legal caretaker and diagnosed with SARS-CoV-2 infection were enrolled after informed consent.

Exclusion criteria: Pregnant children, those with dental infection, trauma to head or neck, active seizures or psychiatric complaints were excluded.

### 4.4. Data and Sample Collection

Saliva samples were obtained after oral rinse by placing a highly absorbent swab in the sub-lingual and parotid regions for approximately 10–20 s using standard kits (Catalog #: ORE-100, Genotek, Kanata, ON, Canada), allowing us to collect pooled saliva from the parotid and sublingual regions. To enhance collection of exosomal RNA, which constitutes the majority of saliva microRNA, care was taken to avoid scraping the buccal, periodontal or enamel surfaces [52]. The samples were immediately placed in RNA stabilizing solution and shipped via priority mail at room temperature to the Penn State Genome Sciences Core Facility for processing. As per manufacturer’s instructions, care was taken to ensure temperature stability. Study data were collected by trained personnel and entered into REDCap [(v 11.4.4), hosted at Wayne State University, Detroit, Michigan, USA [53,54].

### 4.5. RNA Processing

RNA was extracted from each sample using a standard protocol from the miRNeasy Kit (Qiagen, Germantown, MD, USA; Catalog #217004), yielding an average of 927 ng/sample (range: 200–3740 ng). RNA quality was assessed with an Agilent Bioanalyzer (Illumina, San Diego, CA, USA), and sequencing libraries were prepared using a miRNA library kit (Qiagen) at the Penn State Genome Sciences Core Facility. RNA sequencing was performed at a targeted depth of 7.5 million, 30 base, single-end reads per sample (NovaSeq 6000, Illumina). Quantification of miRNAs was performed in the GeneGlobe Data Analysis Center (Qiagen; Catalog # 331502). Adapters were trimmed from the 3′ end, and reads <16 bp or reads without adapters were discarded. Unique reads were aligned to the human genome (hg38) using miRBase mature (V21) and the bowtie algorithm, where up to two mismatches were tolerated. Aligned reads were quantile normalized and each miRNA feature was mean-center scaled (divided by feature standard deviation). Features (miRNAs) were filtered based on abundance (read counts ≥ 10 in ≥10% of samples) and interquartile range, and samples were filtered based on total miRNA alignment (raw reads > 2 × 10^4^) prior to analysis. The samples were examined for sphericity with two-dimensional principal components analysis. Samples outside the 95 % confidence interval were re-analyzed.

### 4.6. Statistical Analysis

Wilcoxon rank sum testing was used to compare miRNAs between the severe and non-severe groups. The miRNAs with ≥log2 fold change and Benjamini–Hochberg adjusted *p* < 0.05 were considered significantly different between groups. A two-dimensional partial least squares discriminant analysis was used to visualize differences in total miRNA profiles between severe and non-severe cases. Biologic relevance of the top miRNAs was explored in DIANA miRpath software (v3), using the micro-T-CDS algorithm (threshold 0.80 for the log2 fold change, *p* < 0.05) to identify gene ontology pathways with over-represented miRNA targets on Fisher chi-square testing (adj *p* < 0.05). This prospective study assumed that 20% of the participants would have severe outcomes. The study planned to perform miRNA-seq on 200 participants. With an average of at least 20 reads per million alignment and coefficient of variation of 0.4 from variability within each group, the minimum detectable fold change is 1.419 for a sample size of 40 versus 160 in the severe and non-severe groups [55].

## 5. Conclusions

Salivary miRNA levels are perturbed in children with severe COVID-19 compared with those with non-severe infections, with majority of the miRNAs being down regulated. Significantly lowered levels of miR-4495, miR-296-5p, miR-548ao-3p and miR-1273c were noted in the saliva of children with severe disease. All four miRNAs displayed pathway enrichment relevant to SARS-CoV-2 infection including viral processing and host immune response. Further studies are required to validate and define the role of salivary miRNAs as potential biomarkers of severe COVID-19 in children.

## Figures and Tables

**Table 1:** Participant Characteristics.

	All (*n* = 197)	Severe (*n* = 45)	Non-Severe (*n* = 152)
Age (Years), Mean (SD) *	7.5 (5.9)	9.4 (5.9)	6.9 (5.7)
Female Sex, *n* (%)	108 (54.8%)	20 (44.4%)	88 (57.9%)
Race, *n* (%)			
American Indian or Alaskan Native	0 (0%)	0 (0%)	0 (0%)
Asian	2 (1.0%)	0 (0%)	2 (1.3%)
Black or African American	126 (64.0%)	31 (68.9%)	95 (62.5%)
White	54 (27.4%)	10 (22.2%)	44 (28.9%)
Other	6 (3.0%)	2 (4.4%)	4 (2.6%)
Unknown	9 (4.5%)	2 (4.4%)	7 (4.6%)
Hispanic Ethnicity, *n* (%)	14 (7.1%)	4 (8.8%)	10 (6.6%)
Public Insurance, *n* (%)	144 (73.1%)	33 (73.3%)	111 (73.0%)
History of Asthma, *n* (%)	36 (18.2%)	8 (17.8%)	28 (18.4%)
History of Diabetes, *n* (%) *	7 (3.6%)	4 (8.8%)	3 (2.0%)
Immunosuppressed, *n* (%) *	2 (1.0%)	2 (4.4%)	0 (0%)
Body Mass Index (kg/m^2^), Mean (SD) *	24.8 (10.8)	28.4 (13.1)	21.9 (7.6)
Received COVID Vaccination, *n* (%)	6 (3.0%)	1 (2.2%)	5 (3.3%)

* Standard deviation.

## Data Availability

miRNA data used in this study are contained within the supplementary material (Supplement Table S1-Differentially expressed miRNAs and Supplement Table S2-Gene ontology pathways).

## Supplementary Materials

The following supporting information can be downloaded at: https://www.mdpi.com/article/10.3390/ijms24098175/s1.
