# Supplementary material for: Saliva microRNA Profile in Children with and without Severe SARS-CoV-2 Infection
Source: Int J Mol Sci. 2023 May 3;24(9):8175. doi: 10.3390/ijms24098175 (PMC10179619; doi:10.3390/ijms24098175)
Supplement: Supplementary file 1 [file ijms-24-08175-s001.zip › Supplemental Table S1 Differentially expressed miRNAs.pdf]

microRNA	FC	log2(FC)	p.ajusted	% Present	Mean Count
hsa-miR-4495	0.19384	-2.3671	9.41E-09	100	3537.769
hsa-miR-296-5p	0.20192	-2.3081	4.97E-07	100	1097.779
hsa-miR-548ao-3p	0.31594	-1.6623	1.09E-05	97.98995	153.809
hsa-miR-1273c	0.31251	-1.678	4.62E-05	98.99497	384.4271
hsa-miR-498	0.16548	-2.5953	0.000439	98.99497	298.5678
hsa-miR-616-5p	0.40931	-1.2887	0.000515	100	3156.618
hsa_piR_001928/gb/DQ572564/Homo	0.35087	-1.511	0.001287	81.90955	95.19095
hsa_piR_009518/gb/DQ582846/Homo	0.40585	-1.301	0.00142	90.95477	186.2362
hsa-miR-4472	0.33642	-1.5717	0.00142	99.49749	192.6734
hsa-miR-6879-3p	0.44743	-1.1603	0.001653	99.49749	145.1256
hsa-miR-548m	0.3592	-1.4772	0.002367	97.98995	101.7739
hsa-miR-1270	0.34708	-1.5266	0.002676	99.49749	267.5377
hsa-miR-4513	2.218	1.1492	0.002752	91.9598	42.9598
hsa-miR-6878-3p	0.3998	-1.3227	0.004068	99.49749	179.4774
hsa-miR-6715b-3p	2.0014	1.001	0.004421	96.98492	43.04523
hsa-miR-6856-5p	0.37045	-1.4326	0.004467	100	169.0804
hsa-miR-874-3p	2.4697	1.3043	0.005694	98.49246	55.74372
hsa-miR-7152-5p	0.36713	-1.4457	0.005895	100	186.8693
hsa-miR-516b-3p	0.47861	-1.0631	0.006219	96.48241	55.85427
hsa_piR_016945/gb/DQ593356/Homo	2.5562	1.354	0.006427	96.48241	114.8191
hsa-miR-24-1-5p	0.43562	-1.1989	0.007437	98.99497	80.30653
hsa-miR-6728-5p	3.034	1.6012	0.007775	99.49749	165.1407
hsa-miR-5690	0.48792	-1.0353	0.009766	100	214.9196
hsa-miR-187-5p	0.49792	-1.006	0.010479	100	88.62312
hsa-miR-149-3p	2.591	1.3735	0.013385	98.49246	70.72362
hsa-miR-6895-3p	0.49994	-1.0002	0.013882	98.49246	95.50251
hsa-miR-3193	0.38145	-1.3904	0.016193	98.99497	287.5327
hsa-miR-4743-3p	0.45896	-1.1236	0.01665	97.98995	52.1206
hsa-miR-6812-3p	2.5	1.3219	0.016924	95.9799	50.44724
hsa-miR-3192-3p	0.44479	-1.1688	0.019018	100	187.8894
hsa-miR-4453	0.37022	-1.4335	0.020932	97.98995	106.3518
hsa-miR-4506	0.42712	-1.2273	0.021386	96.98492	44.03015
hsa-miR-4537	0.46638	-1.1004	0.021386	98.99497	203.7186
hsa-miR-4423-3p	0.33604	-1.5733	0.02258	97.48744	102.995
hsa-miR-3157-3p	0.4705	-1.0877	0.025754	97.48744	97.11055
hsa-miR-202-3p	0.41219	-1.2786	0.025754	99.49749	178.0101
hsa-miR-33a-3p	4.26	2.0908	0.026021	97.98995	55.54271
hsa-miR-6762-3p	0.49678	-1.0093	0.033361	99.49749	92.74874
hsa-miR-6747-3p	2.0515	1.0367	0.034772	95.9799	55.38191
hsa-miR-6511b-3p	0.42862	-1.2222	0.036275	100	176.598
hsa-miR-6844	2.0919	1.0648	0.040381	93.46734	53.42211

hsa-miR-4478	2.3676	1.2434	0.043618	99.49749	130.9799
hsa-miR-188-3p	2.4795	1.3101	0.044143	99.49749	79.28141
