# Supplementary material for: Saliva microRNA Profile in Children with and without Severe SARS-CoV-2 Infection
Source: Int J Mol Sci. 2023 May 3;24(9):8175. doi: 10.3390/ijms24098175 (PMC10179619; doi:10.3390/ijms24098175)
Supplement: Supplementary file 1 [file ijms-24-08175-s001.zip › Supplemental Table S2- Gene ontology pathways.pdf]

GO Category	p-value	#genes	#miRNAs
organelle	1.11E-38	664	4
cellular nitrogen compound metabolic process	1.55E-23	342	4
ion binding	2.10E-19	407	4
biosynthetic process	4.68E-15	282	4
cellular protein modification process	1.05E-11	175	4
protein binding transcription factor activity	1.39E-08	52	3
Fc-epsilon receptor signaling pathway	1.55E-06	21	4
nucleic acid binding transcription factor activity	5.91E-06	79	4
enzyme binding	5.91E-06	98	4
gene expression	7.70E-06	46	2
intrinsic apoptotic signaling pathway	2.07E-05	15	2
nucleoplasm	2.86E-05	90	3
molecular_function	4.66E-05	1011	4
neurotrophin TRK receptor signaling pathway	0.00011209	24	4
positive regulation of protein insertion into mitochondrial membrane involved in apoptotic signaling pathway	0.00013167	9	2
cell death	0.00016925	71	3
cellular_component	0.00034288	1018	4
viral process	0.00043652	36	3
cytosol	0.00060609	177	4
symbiosis, encompassing mutualism through parasitism	0.00071579	39	3
phosphatidylinositol-mediated signaling	0.00339049	17	4
catabolic process	0.00419801	119	3
biological_process	0.00521679	982	4
nervous system development	0.00571457	41	3
transcription, DNA-templated	0.00702432	167	4
cellular lipid metabolic process	0.01053601	15	2
protein complex	0.01496437	224	4
transforming growth factor beta receptor signaling pathway	0.01892793	21	2
small molecule metabolic process	0.02395639	132	4
transcription factor binding	0.02544714	51	4
Fc-gamma receptor signaling pathway involved in phagocytosis	0.02990586	9	1
response to stress	0.03227533	135	4
